# Associations of Antioxidant Enzymes with the Concentration of Fatty Acids in the Blood of Men with Coronary Artery Atherosclerosis

**DOI:** 10.3390/jpm11121281

**Published:** 2021-12-02

**Authors:** Viktoriya S. Shramko, Eugeniia V. Striukova, Yana V. Polonskaya, Ekaterina M. Stakhneva, Marina V. Volkova, Alexey V. Kurguzov, Elena V. Kashtanova, Yuliya I. Ragino

**Affiliations:** 1 Research Institute of Internal and Preventive Medicine, Branch of the Institute of Cytology and Genetics, Siberian Branch of Russian Academy of Sciences (IIPM–Branch of IC&G SB RAS), 175/1 B. Bogatkova Str., 630089 Novosibirsk, Russia; stryukova.j@mail.ru (E.V.S.); yana-polonskaya@yandex.ru (Y.V.P.); stahneva@yandex.ru (E.M.S.); marina_volkova91@mail.ru (M.V.V.); elekastanova@yandex.ru (E.V.K.); ragino@mail.ru (Y.I.R.); 2 Federal State Budgetary Institution “National Medical Research Center Named Academician E.N. Meshalkin”, Ministry of Health of the Russian Federation, Rechkunovskaya Str., 15, 630055 Novosibirsk, Russia; aleksey_kurguzov@mail.ru

**Keywords:** coronary atherosclerosis, ischemic heart disease, saturated fatty acids, monounsaturated fatty acids, polyunsaturated fatty acids, oxidative stress, superoxide dismutase, catalase, glutathione peroxidase

## Abstract

Objective: To identify associations of fatty acids (FAs) with the antioxidant enzymes in the blood of men with coronary atherosclerosis and ischemic heart disease (IHD). Methods: The study included 80 patients: control group—20 men without IHD, the core group—60 men with IHD. The core group was divided into subgroups: subgroup A—with the presence of vulnerable atherosclerotic plaques, subgroup B—with the absence of vulnerable atherosclerotic plaques. We analyzed the levels of FAs, free radicals, superoxide dismutase (SOD), catalase (CAT), and glutathione peroxidase (GPx) in the blood. Results. Patients with IHD, compared with the control group: (1) had higher levels of SOD, CAT, myristic, palmitic, palmitoleic, and octadecenoic FAs; (2) had lower levels of GPx, α-linolenic, docosapentaenoic, docosahexaenoic, and arachidonic FAs. In subgroup A there were found: (1) negative associations of SOD—with linoleic, eicosatrienoic, arachidonic, eicosapentaenoic, docosapentaenoic and docosahexaenoic FAs, positive associations—with palmitic acid; (2) positive correlations of CAT level with palmitoleic and stearic acids; (3) negative associations between of GPx and palmitic, palmitoleic, stearic and octadecenoic FAs. Conclusions: Changes in the levels of antioxidant enzymes, and a disbalance of the FAs profile, probably indicate active oxidative processes in the body and may indicate the presence of atherosclerotic changes in the vessels.

## 1. Introduction

It is widely acknowledged that atherosclerosis is a pathophysiological process that leads to the development of ischemic heart disease (IHD) [1,2]. According to data for 2013, the total incidence of IHD increased by 13.25% over 10 years, and the number of deaths amounted to 7.4 million people, which is a third of all deaths [3,4,5].

Currently, there is a unanimous opinion that oxidative stress plays a significant role in the pathogenesis of atherosclerosis [6]. A disbalance between pro-oxidants and the antioxidant defense of the body leads to the activation of the inflammatory signal and mitochondrial-mediated apoptosis, which, in turn, contributes to the occurrence and development of IHD [7]. Superoxide dismutase (SOD), catalase (CAT), and glutathione peroxidase (GPx) are some of the key enzymes of the antioxidant system.

The SOD is the most active enzyme against reactive oxygen species (ROS) in the myocardium [8]. Due to its antioxidant and anti-apoptotic properties, as well as the ability to inhibit cell hypertrophy, SOD plays a potential protective role against the occurrence of atherosclerosis [9]. However, it should be noted that the role of SOD in preventing free radical oxidation is not entirely obvious. Despite the high specificity of the enzyme, under certain conditions, SOD can interact with H_2_O_2_ and play the role of a pro-oxidant [10]. The CAT is a hydroperoxidase. CAT can interfere with apoptosis by attenuating the H_2_O_2_ signal, and thereby increase the life span of the cell [11,12]. Lei et al. [13] reported that overexpression of CAT protects against cardiovascular dysfunction. The GPx is a selenoprotein enzyme that protects the body from oxidative damage and supports vascular homeostasis [14,15]. GPx deficiency is associated with an increase in the number of oxidants associated with peroxide and subsequent chronic diseases, such as atherogenesis [16] and cardiovascular events [17].

There is evidence that changes in the level of fatty acids (FAs) can disrupt the redox state of cells, not only by increasing the production of ROS, but also by reducing the activity of antioxidant enzymes [18]. Accordingly, FAs are probably one of the physiological factors controlling oxidative stress [19]. Therefore, in our study, we decided to evaluate the relationship of the antioxidant system indicators with the FA concentration in the blood in coronary atherosclerosis.

## 2. Materials and Methods

The study was conducted in the framework of the research work program “IIPM–branch of IC&G SB RAS” jointly with the Federal State Budgetary Institution “National Medical Research Center named after Academician E.N. Meshalkin” of the Ministry of Health of the Russian Federation, as well as with the Novosibirsk Institute of Organic Chemistry SB RAS. The present study has a “descriptive” design.

The local Ethics Committee of the Research Institute of Internal and Preventive Medicine—Branch of the Institute of Cytology and Genetics, Siberian Branch of Russian Academy of Sciences approved the study (protocol №2, approval on 3 July 2017). Each patient gave written informed consent to participate in the study and for the processing of their data.

### 2.1. Subjects

The study included 80 people. The core group consisted of 60 men with coronary angiographically verified atherosclerosis of the coronary arteries, with IHD, with stable angina pectoris and without acute coronary syndrome (ACS), who was admitted to the clinic of the E. N. Meshalkin National Medical Research Center of the Ministry of Health of the Russian Federation for coronary bypass surgery (CBS). The age of the patients was 59.43 ± 7.38 years.

Inclusion criteria were male gender, diagnosis of IHD, verified by coronary angiography data, history of a previous myocardial infarction (MI) or episodes of stable angina pectoris (more than six months before admission), documented by the description of the clinical picture, the results of ECG and biochemical blood tests.

Exclusion criteria were being of female gender, clinically significant severe concomitant pathology in the acute stage (chronic infectious and inflammatory diseases, kidney failure, respiratory failure, liver failure), known active oncological diseases, hyperparathyroidism, toxic damage by heavy metals, and taking steroid and non-steroidal anti-inflammatory drugs.

As a control group, 20 men were taken from a population sample of Novosibirsk without IHD, comparable to the core group in terms of age and body mass index (BMI). Inclusion criteria were being of male gender, the absence of IHD, being verified by the results of a complete clinical and instrumental examination, and the absence of therapy with cardiovascular drugs. The screening of the control group was carried out based on IIPM—a branch of ICIG SB RAS. Table 1 presents the characteristics of patients in the core and control groups.

According to the results of the histological analysis of intima-media pieces, 45% of the patients in the core group had vulnerable atherosclerotic plaques in coronary arteries (subgroup A), whereas in 55% of patients, no vulnerable atherosclerotic plaques were detected (subgroup B). Table 2 presents the clinical-biochemical characteristics of patients of the studied subgroups.

### 2.2. Methods

#### 2.2.1. Histological Analysis

During CBS, patients underwent endarterectomy of the coronary arteries strictly according to intraoperative indications. All endarterectomy samples were sent for histological analysis to the pathomorphological laboratory of the “National Medical Research Center named after Academician E.N. Meshalkin”. Each endarterectomy material containing the intima-media of the coronary arteries was divided longitudinally and transversely into 3–5 fragments for histological studies. The pathologist performed a macroscopic description of the samples (the prevalence of atherosclerotic plaque, the degree of the artery lumen narrowing, hemorrhages in the structures of atherosclerotic plaque, calcification sites, blood clots) and standard hematoxylin-eosin and Van Gieson staining, following which histological analysis of intima-media fragments was made using an Axiostar Plus binocular microscope.

A vulnerable atherosclerotic plaque was differentiated according to the following criteria: the thickness of the fibrous covering being less than 65 microns, infiltration by macrophages, and T-lymphocytes (more than 25 cells in the field of view of 0.3 mm), as well as a large lipid nucleus (more than 40%) [20].

#### 2.2.2. Fatty Acids Analysis

The Laboratory of Environmental Research and Chromatographic Analysis of the Novosibirsk Institute of Organic Chemistry named after N. N. Vorozhtsov, SB RAS, carried out the determination of the qualitative and quantitative composition of FAs. After extraction and methanolysis, the composition of FAs was studied in all samples using high-efficiency capillary gas-liquid chromatography on an Agilent Technologies (AT) 6890N chromatograph with a flame ionization detector and chromatography-mass spectrometry on an AT 6890N chromatograph with an AT 5975N mass-selective detector. Studied the content of saturated FAs: myristic (C14:0), palmitic (C16:0), stearic (C18:0) and unsaturated FAs (essential unsaturated fatty acids): palmitoleic (C16:1, ω-7), octadecenoic (C18:1, ω-9), eicosenoic (C20:1, ω-9), linoleic (C18:2, ω-6), α-linolenic (C18:3, ω-3), γ-linolenic (C18:3, ω-6), eicosatrienoic (C20:3, ω-6), eicosapentaenoic (C20:5, ω-3), docosapentaenoic (C22:5, ω-3), docosahexaenoic (C22:6, ω-3) and arachidonic (C20:4, ω-6).

#### 2.2.3. Biochemical Studies

Peripheral (venous) blood sampling was performed in all patients included in the study (for patients of the core group before the CBS), at least 12 h after the last meal with a night’s rest, as well as abstinence from physical exertion, stressful situations, physiotherapy, medication, alcohol use, smoking, and fatty foods. The blood was kept at room temperature for 30 min, then centrifuged at a speed of 1900 g for 15 min. Then, the blood specimens were separated into plasma or serum, and stored at −70 °C until analysis.

The Laboratory of Clinical Biochemical and Hormonal Studies of Therapeutic Diseases of IIPM–branch of IC&G SB RAS carried out the biochemical studies. The concentration of total cholesterol, triglycerides, and high-density lipoprotein (HDL) was determined by the enzymatic method using the Thermo Fisher Scientific kits on the Konelab Prime 30i biochemical analyzer (Thermo Fisher Scientific, Finland). The levels of LDL were calculated using the Friedwald formula. The level of lipid peroxidation products (LPO) in LDL isolated from blood serum was evaluated by the content of TBA-reactive products in LDL by the fluorimetric method on the Versafluor spectrofluorimeter, Bio-Rad. The free radical detection test (FORT) was evaluated using the FORM Plus CR3000 analyzer (Callegary, Italy). The FORT colorimetric test is based on the ability of transition metals to catalyze the cleavage of hydroxyperoxides (ROOH) with the formation of free radicals. The reading of the FORT test results was based on a linear kinetic reaction. The absorption value was automatically converted into conventional units, called units H2O2. The CAT and GPx1 concentrations were evaluated by enzyme immunoassay (ELISA) using standard ELISA test systems (Cloud-Clone Corp., USA), Hu Cu/ZnSOD (SOD1) concentrations were evaluated using ELISA test systems (Bender MedSystems, Austria), on the MultiscanEX ELISA analyzer (Thermo Labsystem, Finland). These ELISA methods were designed for the quantitative determination of enzymes in serum, plasma, cell lysates, and so forth.

#### 2.2.4. Statistical Methods

Statistical processing of the results was carried out using the IBM SPSS Statistics software package (version 20.0). The Kolmogorov–Smirnov test was used to assess the distribution of quantitative features. A comparative study of clinical and anamnestic characteristics in the groups was carried out using Student’s t-test. In the text, these characteristics are presented in the form of an arithmetic mean (M) and a standard deviation (SD). In the presence of an abnormal distribution of signs (study of fatty acids and antioxidant enzymes), the nonparametric Mann–Whitney U-test was used (for two independent groups). The obtained data are presented in the form of a median (Me) with the interquartile range—the 25th and 75th percentiles. The Spearman rank correlation coefficient (r_s_) was used to analyze the dependence of quantitative features of sample data from aggregates. Multiple logistic regression was used to assess the probability of the unstable atherosclerotic plaques presence in the coronary arteries. In the Table, the results are presented as a ratio of chances (OR) with a 95% confidence interval for OR. The difference in proportions and the nature of associations was evaluated by the Pearson criterion χ2. The criterion of the statistical reliability of the obtained data was set at the level of *p* < 0.05.

## 3. Results

When assessing the levels of the lipid spectrum, men with IHD, compared with the control group, had increased levels of triglycerides by 1.7 times (*p* = 0.005) and decreased levels of HDL by 1.45 times (*p* = 0.010). The level of LPO increased by 1.4 times (*p* = 0.053) (Table 1).

When analyzing the clinical characteristics of the subgroups, it was revealed that a history of MI occurred in 81% of cases in subgroup A and 61% in subgroup B, but there was no statistically significant difference (Table 2). The angina pectoris of functional class (FC) III was established in 70% of men from subgroup A, which differed from subgroup B by 1.7 times (*p* = 0.031). To describe the severity of the chronic heart failure (CHF) symptoms was used FC classification following the criteria proposed by the New York Association of Cardiologists (NYHA) [21]. In subgroup A, the number of people with CHF of FC III was 92%, which significantly differed from subgroup B, where the number of people with CHF of FC III was 69% (*p* = 0.027). According to the results of coronary angiography, hemodynamically significant severe stenosis was found in all patients (>70% for all coronary arteries, except for the left main coronary artery, where stenosis > 50% is considered significant) [22].

**Figure 1:**
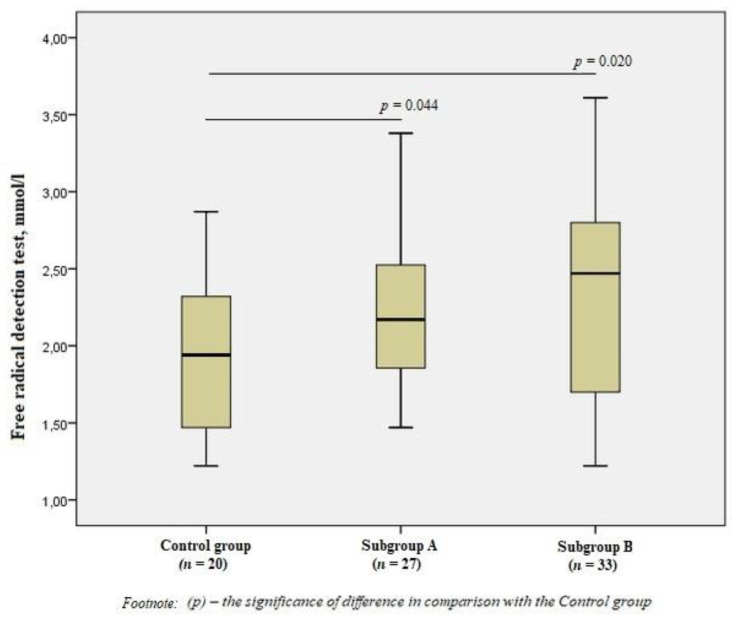
Free radical detection test in the group of men without ischemic artery disease (Control group), in the subgroup of men with ischemic artery disease and the presence of vulnerable atherosclerotic plaques in the coronary arteries (Subgroup A), and the subgroup of men with ischemic artery disease and the absence of vulnerable atherosclerotic plaques (Subgroup B), Me (25%; 75%). *p*—the significance of difference in comparison with the Control group.

When evaluating free radicals in the subgroups, we revealed significant differences from the Control group values (*p* < 0.05), but there were no significant differences between patients with the presence/absence of vulnerable atherosclerotic plaques (Figure 1).

**Figure 2:**
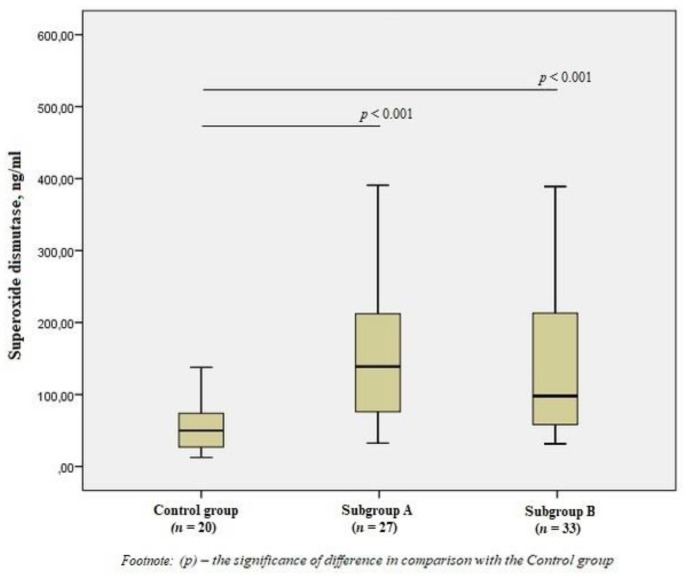
The content of superoxide dismutase in the blood of men without ischemic artery disease (Control group), men with ischemic artery disease and the presence of vulnerable atherosclerotic plaques in the coronary arteries (Subgroup A) and men with ischemic artery disease and the absence of vulnerable atherosclerotic plaques (Subgroup B), Me (25%; 75%). *p*—the significance of difference in comparison with the Control group.

When studying antioxidant enzymes, there was an increase in the level of SOD1 in subgroup A by 2.8 times and subgroup B by 2.5 times compared to the control group (*p* < 0.001) (Figure 2), however, there was no statistical difference between the subgroups.

**Figure 3:**
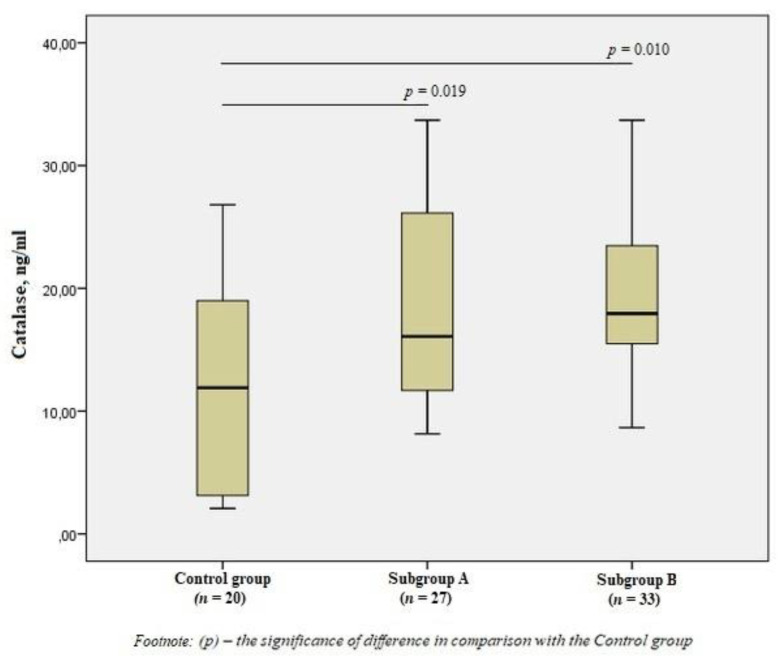
The blood catalase levels in the group of men without ischemic artery disease (Control group), in the subgroup of men with ischemic artery disease and the presence of vulnerable atherosclerotic plaques in the coronary arteries (Subgroup A) and the subgroup of men with ischemic artery disease and the absence of vulnerable atherosclerotic plaques (Subgroup B), Me (25%; 75%). *p*—the significance of difference in comparison with the Control group.

There was a significant increase in the level of CAT in subgroup A by 1.3 times (*p* = 0.019) and subgroup B by 1.5 times (*p* = 0.010) compared to the control group (Figure 3), but there was no difference in the content of CAT between the subgroups (*p* > 0.05).

**Figure 4:**
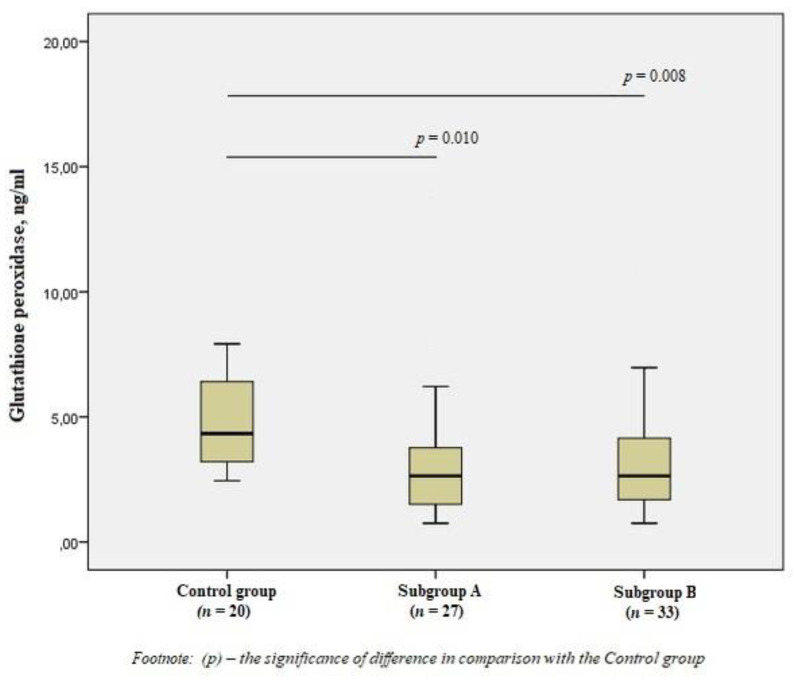
The concentrations of glutathione peroxidase in the blood of men without ischemic artery disease (Control group), men with ischemic artery disease and the presence of vulnerable atherosclerotic plaques in the coronary arteries (Subgroup A) and men with ischemic artery disease and the absence of vulnerable atherosclerotic plaques in the coronary arteries (Subgroup B), Me (25%; 75%). *p*—the significance of difference in comparison with the Control group.

When studying GPx1, we obtained the opposite results—a significant decrease in the level of the enzyme in patients of the subgroups by 1.7 times compared with the control group (*p* < 0.01) (Figure 4).

**Figure 5:**
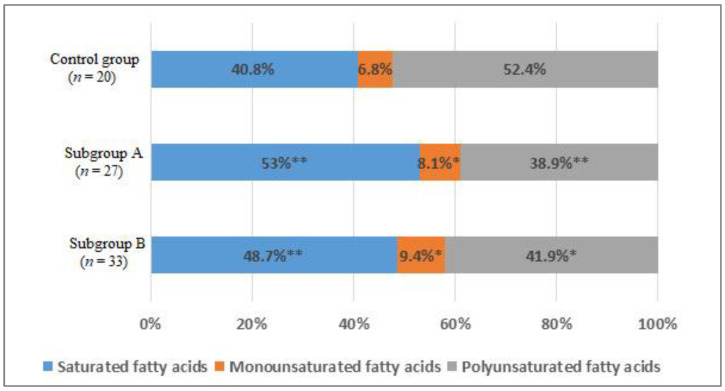
The percentage of saturated (SFAs), monounsaturated (MUFAs), and polyunsaturated fatty acids (PUFAs) of the total content of fatty acids in the blood of men without ischemic artery disease (Control group), men with ischemic artery disease and the presence of vulnerable atherosclerotic plaques in the coronary arteries (Subgroup A) and men with ischemic artery disease and the absence of vulnerable atherosclerotic plaques in the coronary arteries (Subgroup B), %. *—*p* < 0.05, **—*p* < 0.01, *p*—the significance of difference in comparison with the Control group.

When studying the distribution of FAs in blood serum, the comparative analysis showed a statistically significant increase in the total content of the saturated FAs (SFAs) fraction by 12% in subgroup A (*p* < 0.01) and by 8% in the subgroup B (*p* < 0.05) compared with the control group (Figure 5).

Mainly due to an increase in the level of myristic acid by 1.3 and 1.8 times, respectively (*p* < 0.05), and the level of palmitic acid by 1.6 and 1.3 times, respectively (*p* < 0.05) (Table 3).

There was an increase in the total content of monounsaturated FAs (MUFAs) (*p* < 0.05) in subgroups compared with the group of men without IHD (Figure 5). An increase in the level of palmitoleic acid (1.6 times, *p* < 0.01) and octadecenoic acid (1.3 times, *p* < 0.05) was found (Table 3).

In addition, there was a decrease in the total content of polyunsaturated FAs (PUFAs) by 13.5% in subgroup A (*p* < 0.01) and by 10.5% in subgroup B (*p* < 0.05) (Figure 5). The level of ω-6 arachidonic acid in subgroup A decreased by 1.35 times, in subgroup B by 1.5 times (*p* < 0.05). The content of ω-3 docosapentaenoic acid in subgroup A was lower in 1.55 times, in subgroup B—1.9 times (*p* < 0.05); docosahexaenoic acid—1.4 and 1.7 times, respectively (*p* < 0.05). The level of α-linolenic acid only in subgroup A differed significantly from the values of the control group 1.6 times (*p* < 0.05) (Table 3).

The analysis of the FAs content did not reveal statistically significant differences between the subgroups.

Table 4 shows the results of the correlation analysis between the content of FAs and the antioxidant system enzymes in men of subgroup A.

Table 5 shows the results of the correlation analysis between the content of FAs and the antioxidant system enzymes in men of subgroup B.

Significant inverse associations were established between the indicators of FORT and PUFAs—arachidonic and docosahexaenoic acids (*p* < 0.05) from the studied spectrum of FAs in the blood of patients with vulnerable plaques. When studying the indicators of the antioxidant system, negative correlations of the SOD level with PUFAs—linoleic, arachidonic, eicosapentaenoic, eicosatrienoic, docosapentaenoic, and docosahexaenoic (*p* < 0.05), and positive associations with palmitic acid (*p* < 0.01) were revealed. The positive correlations of the CAT level with the content of palmitoleic and stearic acids (*p* < 0.05) were revealed. The negative correlations were established between the concentrations of GPx and palmitic, palmitoleic, octadecenoic, and stearic acids (*p* < 0.05).

In men with atherosclerosis of the coronary arteries and the absence of vulnerable atherosclerotic plaques, the results of the correlation analysis were markedly different. The negative associations were established between FORT and all the PUFAs. For eicosenoic acid, a direct relationship was found; for eicosatrienoic, an inverse relationship with the SOD level was found (*p* < 0.05). The CAT levels are inversely dependent on the level of eicosatrienoic acid and directly on the level of octadecenoic acid (*p* < 0.05). In the subgroup of patients without vulnerable plaques, no significant associations with GPx were found.

To assess the probability of atherosclerotic changes in the coronary arteries depending on the content of antioxidant enzymes (Table 6), we used multivariate logistic regression analysis. The presence of vulnerable atherosclerotic plaques in the coronary arteries toward their absence was used as a dependent variable. The content of free radicals and all the antioxidant enzymes studied by us was used as independent variables.

The results showed that an increase in the content of SOD and CAT in the blood is associated with a relative risk of having vulnerable atherosclerotic plaques in the coronary arteries.

## 4. Discussion

The study of the lipid profile, including the FAs profile, is a promising direction in clinical diagnostics for the early detection of persons with a high risk of CVD.

We have obtained data on the increase in the level of myristic and palmitic SFAs in the blood of patients with IHD, which is consistent with the data in the literature. Lausada et al. [23] identified that in the blood plasma of patients with IHD, there is a significant increase in SFAs, mainly due to palmitic acid, and, to a lesser extent, oleic and stearic acids, compared with the control groups. In a large-scale study, CIRCS [24], the authors found that high levels of saturated myristic and palmitic FAs had the most adverse effect on the development of CVD. Björck et al. [25], in their study, concluded that an increase in the consumption of SFAs affects the growth of total cholesterol in the blood and, therefore, positively influenced mortality from IHD. Conversely, even a moderate decrease in the SFA content and replacing them with PUFAs in the diet significantly reduced the risk of developing CVD [26].

Therefore, the determination of the content of SFAs in the blood, especially palmitic and myristic, can be used in the clinical diagnosis of atherosclerosis-associated diseases.

When studying the content of MUFAs, we obtained a significant increase in the content of palmitoleic and octadecenoic FAs in the group of patients with atherosclerosis, which probably indicates that these FAs are associated with CVD risk. Several researchers have found an association between palmitoleic acid and cardiovascular risk factors [27]. Chei et al. [24] found that high levels of palmitoleic acid increased the risk of IHD. On the contrary, data from some controlled randomized trials have shown that MUFAs have a beneficial effect on the profile of lipoproteins in the blood, and as a consequence, on reducing the risk of CVD [28]. The results obtained may cast doubt on the supposed positive effects of MUFAs in the body, particularly in IHD.

Increasingly more studies are being published showing the influence not only of SFAs, and some MUFAs on CVD, but also the effect of PUFAs [26]. According to our data, in the core group of men with IHD, the content of very long-chain omega-3 PUFAs (such as docosapentaenoic and docosahexaenoic FAs) and arachidonic omega-6 PUFA was significantly lower when compared with the control group. That suggests that even in the subgroups of PUFAs, specific individual FAs may have a different impact on the risk of developing IHD [24]. In addition, in recent years, the complex biochemistry of eicosanoids has become clearer, so the very class of omega-6 PUFAs can no longer be so simply considered as pro-inflammatory [29].

On the one hand, obtained high levels of SFAs and MUFAs can probably block the bioavailability of PUFAs for cells and form a deficiency of PUFAs in cells, which contributes to the development of the atherosclerotic process [30]. On the other hand, the high degree of unsaturation of PUFAs makes these FAs very susceptible to oxidation, which makes them “harmful” and leads to an increase in the number of oxidative molecules that trigger inflammatory reactions [19,31].

Oxidative stress occurs due to an imbalance between the production of ROS and a decrease in the overall antioxidant capacity. We studied the levels of free radicals and antioxidant indicators as markers of oxidative stress. Excessive formation of H_2_O_2_ was found in patients with atherosclerosis and, accordingly, a high degree of oxidative damage by free radicals. The reduced content of individual PUFAs in the blood of patients with atherosclerosis, as well as increased lipid peroxidation, confirms the influence of oxidative stress on the pathogenesis of IHD.

Currently, increasingly more attention is being paid to the quantitative assessment of antioxidant enzymes. Under normal conditions, SOD is the first line of defence against oxidative stress [7]. A meta-analysis conducted by Flores-Mateo et al. [32], which included 26 case-control studies, evaluated the relationship between the level of activity of SOD and IHD. In most studies, inverse associations between SOD and the outcome of IHD were found, although they were not always statistically significant [32]. In our study, there was a significant increase in the level of the SOD enzyme in the blood serum of patients with IHD, compared with the control group. Vichova et al. [33], in their work, reported that oxidative stress directly triggers the cascade of inflammation and accelerates the oxidation of lipids containing PUFAs, causing vulnerability of atherosclerotic plaques. This explains the direct relationship between we obtained the SOD level and a lower PUFAs content in patients with vulnerable plaques since the high production of the SOD enzyme is aimed at neutralizing oxidative damage by free radicals.

Whenever ROS are involved, CAT, together with other enzymes, forms a reliable defence of the body [34,35]. Overexpression of CAT has been reported to prevent the stimulation of ROS and protect against cardiovascular dysfunction or injury [14,36]. A study by Gupta et al. [37] showed that in the early stages of IHD, the levels of SOD and CAT increased to protect and prevent lipid peroxidation. Our study also shows an increase in SOD and CAT levels in patients with IHD, despite the prolonged course of the disease. Animal studies have shown that oleic acid (C 18: 1) induces an increase in the level of H_2_O_2_ [38]. In our study, the increased level of CAT was directly related to the content of octadecenoic acid (C 18:1) in the group of patients with atherosclerosis. We suggest that this may be due to excessive production of H_2_O_2_ in the vessels of these patients.

SOD oxidation (mainly by H_2_O_2_) can affect His residues (oxidized to 2-oxo derivatives), and catalase can be oxidatively modified after reaction with H_2_O_2_ or malondialdehyde (which results from the OH radical generated by either the Haber-Weiss or Fenton reactions). It is likely that the measured circulating concentrations of these enzymes may not be strictly related to endogenous antioxidant defences, as the mentioned modifications lead to decreased activity of these enzymes.

The GPx is another intracellular antioxidant, the deficiency of which is associated with atherogenesis [17] and the occurrence of IHD [39]. A meta-analysis, which included 32 case-control studies and two prospective cohort studies, showed there was significant heterogeneity in the direction and magnitude of the relationship between GPx and the outcomes of IHD, although, in most studies, an inverse relationship with IHD was found [32]. In our study, the GPx level in men with atherosclerosis was significantly lower than in the control group. Codoñer-Franch et al. [40] showed that following a diet with a reduced content of SFAs for six months significantly improved the antioxidant ability in the form of increased activity of SOD, CAT, and GPx. In our study, we obtained an inverse association between the GPx level and the SFAs content in patients with vulnerable plaques, which probably indicates a violation of the antioxidant defence system and contributes to the development of cardiovascular complications.

In addition, we wanted to assess the probability of the presence of vulnerable atherosclerotic plaques in the coronary arteries, depending on the content of antioxidant enzymes. According to multivariate regression analysis, it can be assumed that in patients with IHD, high levels of SOD and CAT in the blood are associated with a relative risk of destabilization of atherosclerotic plaques. Therefore, it is necessary to carry out a complex of preventive and therapeutic measures to prevent the development of acute coronary syndrome.

### Limitations

One of the limitations of our study is the small sample size, since our study is a pilot. Our findings need further confirmation in a larger study, with a sufficient sample size to allow meta-analyses to determine specific risk profiles. In addition, frozen biomaterial was used in this work. Therefore, it was decided to determine the concentrations of antioxidant enzymes without studying their activity. Since the measured circulating concentrations do not determine the true antioxidant capacity, and an increased activity may normalize or aggregate the differences that are currently observed, it is necessary to measure activity in follow-up studies. Finally, this type of study cannot demonstrate a causal relationship because it is a descriptive, not an experimental study.

## 5. Conclusions

Changes in the levels of antioxidant enzymes, and a disbalance in the FA profile, may probably indicate active oxidative processes in the body and indicate the presence of atherosclerotic changes in the vessels. In addition, changes in the levels of SOD and CAT in the blood of patients with an already established diagnosis of IHD may indicate the presence of destabilizing (i.e., vulnerable) atherosclerotic plaques in the coronary arteries, which requires further study.

## Figures and Tables

**Table 1:** Characteristics of patients in the core and control groups.

Parameter	Control Group (*n* = 20)	Core Group (*n* = 60)	*p*
Mean age, years (M ± SD)	56.7 ± 2.65	59.4 ± 7.38	0.116
BMI, kg/m^2^ (M ± SD)	27.18 ± 2.49	29.05 ± 4.55	0.266
Systolic BP, mmHg (M ± SD)	130.64 ± 11.47	135.8 ± 11.45	0.204
Diastolic BP, mmHg (M ± SD)	83.33 ± 8.35	82.07 ± 9.64	0.741
Heart rate, beats per minute (M ± SD)	66.13 ± 7.21	68.16 ± 8.38	0.372
Tobacco use (absolute in %)	15%	15%	0.956
Alcohol use (absolute in %)	45%	50%	0.770
Lipid profile (M ± SD):			
Cholesterol, mg/dL	171.6 ± 10.75	202.83 ± 6.76	0.175
Triglycerides, mg/dL	87.45 ± 14.71	153.9 ± 11.38	0.005
HDL, mg/dL	47.6 ± 2.27	32.7 ± 1.17	0.010
LDL, mg/dL	110.5 ± 9.92	131.8 ± 7.14	0.092
Content of LPO in LDL after 30-min oxidation, nM MDA/mg LDL protein	16.43 ± 2.41	22.71 ± 10.25	0.053

Footnote: BMI—body mass index; BP—blood pressure; HDL—high-density lipoprotein; LDL—low-density lipoprotein; LPO—lipid peroxidation products; M—mean; MDA—malonic dialdehyde; SD—standard deviation.

**Table 2:** Clinical-biochemical characteristics of patients of the subgroups.

Parameter	Subgroup A (*n* = 27)	Subgroup B (*n* = 33)	*p*
Mean age, years (M ± SD)	60.5 ± 6.48	58.6 ± 8.03	0.307
BMI, kg/m^2^ (M ± SD)	29.91 ± 3.78	28.35 ± 5.05	0.191
Systolic BP, mmHg (M ± SD)	137.57 ± 12.75	134 ± 10.18	0.222
Diastolic BP, mmHg (M ± SD)	82.82 ± 9.86	81.45 ± 9.58	0.589
Heart rate, beats per minute (M ± SD)	69.63 ± 9.37	66.96 ± 7.4	0.222
Hypertensive disease (absolute in %):	96%	82%	0.362
History of heart attack (absolute in %)	81%	61%	0.082
Angina pectoris (absolute in %): II FC III FC IV FC	7% 70% 11%	21% 42% 12%	0.141 0.031 0.906
Multivessel coronary artery disease (absolute in %)	89%	82%	0.518
Tobacco use (absolute in %)	7%	21%	0.141
Alcohol use (absolute in %)	44%	56%	0.585
Dyslipidemia (absolute in %)	70%	73%	0.844
Lipid profile (M ± SD): Cholesterol, mg/dL	202.5 ± 7.39	203.1 ± 11.01	0.963
Triglycerides, mg/dL	170.3 ± 12.44	171.4 ± 20.14	0.993
HDL, mg/dL	32.5 ± 1.71	33 ± 1.6	0.855
LDL, mg/dL	131.4 ± 6.47	132.1 ± 7.33	0.945
Content of LPO in LDL after 30-min oxidation, nM MDA/mg LDL protein	21.71 ± 10.12	23.59 ± 10.57	0.601
History of type II diabetes (absolute in %)	37%	22%	0.241

Footnote: BMI—body mass index; BP—blood pressure; FC—functional class; HDL—high-density lipoprotein; LDL—low-density lipoprotein; LPO—lipid peroxidation products; M—mean; MDA—malonic dialdehyde; NYHA—New York Heart Association functional classification; SD—standard deviation.

**Table 3:** The content of FAs in the blood of men in Subgroups and in the Control group, Me (25%; 75%), mg/dL.

Fatty Acids	Control Group (*n* = 20)	Subgroup A (*n* = 27)	Subgroup B (*n* = 33)
Saturated fatty acids			
Myristic (C 14:0)	2.57 (1.01; 4.58)	3.37 (2.69; 6.21) *	4.72 (2.24; 5.49) *
Palmitic (C 16:0)	84.68 (37.42; 134.25)	132.66 (81.62; 145.8) *	105.65 (88.93; 142.72) *
Stearic (C 18:0)	29.34 (14.84; 49.39)	38.46 (23.9; 42.59)	33.79 (23.87; 38.79)
Monounsaturated fatty acids			
Palmitoleic (C 16:1, ω-7)	6.4 (2.23; 10.45)	10.13 (5.69; 15.45) **	10.3 (7.35; 12.76) **
Octadecenoic (C 18:1, ω-9)	11.97 (7.1; 15.18)	15.43 (12.78; 19.32) *	16.26 (11.9; 18.44) *
Eicosenoic (C 20:1, ω-9)	1.07 (0.42; 1.77)	1.1 (0.82; 1.32)	1.14 (0.85; 1.56)
Omega-3 polyunsaturated fatty acids			
α-linolenic (C 18:3, ω-3)	5.52 (1.22; 9.31)	3.32 (2.43; 3.51) *	3.86 (2.83; 4.96) *
Eicosapentaenoic (C 20:5, ω-3)	1.97 (1.32; 3.42)	2.3 (1.06; 3.09)	1.56 (0.91; 2.72)
Docosapentaenoic (C 22:5, ω-3)	1.77 (0.9; 2.73)	1.14 (0.62; 2.12) *	0.93 (0.57; 1.83) *
Docosahexaenoic (C 22:6, ω-3)	6.58 (3.38; 10.3)	4.62 (2.5; 8.57) *	3.96 (2.6; 7.12) *
Omega-6 polyunsaturated fatty acids			
Linoleic (C 18:2, ω-6)	110.94 (63.45; 179.86)	97.13 (72.34; 124.78)	96.94 (69.33; 120.92)
Arachidonic (C 20:4, ω-6)	16.65 (11.77; 35.94)	12.3 (8.24; 22.57) *	11.33 (7.67; 19.76) *
γ-linolenic (C 18:3, ω-6)	1.14 (0.75; 4.03)	1.47 (0.75; 2.29)	1.27 (0.56; 1.79)
Eicosatrienoic (C 20:3, ω-6)	5.27 (2.59; 8.12)	5.74 (3.59; 7.29)	4.24 (3.19; 6.74)

Footnote: * (*p* < 0.05); ** (*p* < 0.01) —the significance of difference in comparison with the Control group.

**Table 4:** The associations between the content of FAs and the antioxidant system enzymes in men with ischemic artery disease and the presence of vulnerable atherosclerotic plaques in the coronary arteries (Subgroup A).

Fatty Acids	Rank Coefficients of Spearman Correlation (r_s_)			
	FORT	SOD	CAT	GPx
Palmitic (C 16:0)	—	r = 0.515 *p* = 0.005	—	r = −0.339 *p* = 0.059
Palmitoleic (C 16:1, ω-7)	—	—	r = 0.373 *p* = 0.040	r = −0.331 *p* = 0.060
Stearic (C 18:0)	—	—	r = 0.412 *p* = 0.033	r = −0.375 *p* = 0.039
Octadecenoic (C 18:1, ω-9)	—	—	—	r = −0.372 *p* = 0.041
Linoleic (C 18:2, ω-6)	—	r = −0.430 *p* = 0.025	—	—
Eicosatrienoic (C 20:3, ω-6)	—	r = −0.453 *p* = 0.018	—	—
Arachidonic (C 20:4, ω-6)	r = −0.362 *p* = 0.043	r = −0.407 *p* = 0.035	—	—
Eicosapentaenoic (C 20:5, ω-3)	—	r = −0.464 *p* = 0.015	—	—
Docosapentaenoic (C 22:5, ω-3)	—	r = −0.464 *p* = 0.015	—	—
Docosahexaenoic (C 22:6, ω-3)	r = −0.388 *p* = 0.036	r = −0.549 *p* = 0.003	—	—

Footnote: CAT—catalase; FORT—free radical detection test; GPx—glutathione peroxidase; SOD—superoxide dismutase

**Table 5:** The associations between the content of FAs and the antioxidant system enzymes in men with ischemic artery disease and the absence of vulnerable atherosclerotic plaques in the coronary arteries (Subgroup B).

Fatty Acids	Rank Coefficients of Spearman Correlation (r_s_)		
	FORT	SOD	CAT
Octadecenoic (C 18:1, ω-9)	—	—	r = 0.405 *p* = 0.019
Linoleic (C 18:2, ω-6)	r = −0.448 *p* = 0.009	—	—
α-linolenic (C 18:3, ω-3)	r = −0.390 *p* = 0.033	—	—
γ-linolenic (C 18:3, ω-6)	r = −0.388 *p* = 0.031	—	—
Eicosenoic (C 20:1, ω-9)	—	r = 0.413 *p* = 0.017	—
Eicosatrienoic (C 20:3, ω-6)	r = −0.397 *p* = 0.022	r = −0.414 *p* = 0.017	r = −0.307 *p* = 0.053
Arachidonic (C 20:4, ω-6)	r = −0.512 *p* = 0.002	—	—
Eicosapentaenoic (C 20:5, ω-3)	r = −0.471 *p* = 0.006	—	—
Docosapentaenoic (C 22:5, ω-3)	r = −0.518 *p* = 0.002	—	—
Docosahexaenoic (C 22:6, ω-3)	r = −0.588 *p* = 0.0001	—	—

Footnote: CAT—catalase; FORT—free radical detection test; SOD—superoxide dismutase.

**Table 6:** Results of a multivariate logical regression analysis for predicting coronary artery atherosclerosis by the activity of antioxidant enzymes.

Antioxidant Enzymes	B	Exp(B)	95.0% C.I. for Exp(B)		*p*
			Lower	Upper	
FORT	0.396	1.485	0.428	5.157	0.533
SOD	0.020	1.020	1.003	1.037	0.019
CAT	0.107	1.113	1.001	1.239	0.050
GPx	−0.096	0.908	0.753	1.095	0.314

Footnote: CAT—catalase; C.I.—confidence interval, Exp(B)—odds ratio; FORT—free radical detection test; GPx—glutathione peroxidase; SOD—superoxide dismutase.

## Data Availability

The datasets before and after analysis in this study are available from the corresponding author on reasonable request.
